# Associations of Low-Carbohydrate High-Fat Dietary Patterns with Colorectal Tumor Burden and Gut Microbial Dynamics in an AOM/DSS Mouse Model

**DOI:** 10.3390/ijms27136023

**Published:** 2026-07-04

**Authors:** Jae Hyun Kim, Eun-Kyung Ahn, Hee Kyung Chang, Sook-Ja Kim, Jongsik Kim, Seun Ja Park, Jeonghoon Heo

**Affiliations:** 1 Department of Gastroenterology, Kosin University College of Medicine, Busan 49267, Republic of Korea; kjh8517@kosinmed.or.kr; 2 Department of Molecular Biology and Immunology, Kosin University College of Medicine, Busan 49267, Republic of Korea; ekahn@kosin.ac.kr (E.-K.A.); sjkim@kosin.ac.kr (S.-J.K.); 3 Department of Pathology, Kosin University College of Medicine, Busan 49267, Republic of Korea; changhkg@kosinmed.or.kr; 4 Department of Anatomy, Kosin University College of Medicine, Busan 49267, Republic of Korea; jongsikkim@kosin.ac.kr

**Keywords:** colorectal neoplasms, diet, high-fat, carbohydrate-restricted, gastrointestinal microbiome, lymphocytes

## Abstract

Malignant tumors require substantial energy sources for proliferation, and dietary composition may influence colorectal carcinogenesis through metabolic and microbiome-related mechanisms. This study investigated the association of low-carbohydrate high-fat dietary patterns with macroscopic tumor burden, morphologic inflammatory cell infiltration, and gut microbiome alterations using an azoxymethane/dextran sulfate sodium (AOM/DSS)-induced mouse model of colitis-associated colorectal cancer. Male C57BL/6 mice received AOM followed by three cycles of DSS and were fed a standard diet (SD), high-carbohydrate diet (HCD), low-carbohydrate high-fat lard-based diet (HFL), or low-carbohydrate high-fat coconut oil-based diet (HFC). Body weight, colon length, splenic weight, macroscopic tumor formation, hematoxylin and eosin (H&E)-based inflammatory cell infiltration, and gut microbiome composition were analyzed. The HFL and HFC groups exhibited higher body weights and relatively preserved colon lengths compared with the SD and HCD groups. Tumor number and total tumor size were reduced in the HFL and HFC groups. Total lymphocyte-like inflammatory cell infiltration was not increased in the high-fat diet groups, whereas per-tumor values were interpreted cautiously because they are affected by tumor number. Gut microbiome analysis demonstrated altered microbial composition, increased alpha diversity, and distinct temporal microbial dynamics in the high-fat diet groups. Because the HFL and HFC diets simultaneously changed carbohydrate content, fat content, fat source, and caloric density, these findings should be interpreted as exploratory effects of low-carbohydrate high-fat dietary patterns rather than independent effects of carbohydrate restriction, total fat, or fat source.

## 1. Introduction

Colorectal cancer (CRC) is one of the most common malignancies worldwide, and a leading cause of cancer-related mortality [1,2]. Numerous epidemiological studies have revealed that the onset of CRC is closely related not only to genetic predisposition, but also to environmental factors, particularly dietary habits [1,3]. Diets high in fat and carbohydrates, which are characteristic of Westernized dietary patterns, have been identified as major risk factors that promote the initiation and progression of CRC by inducing chronic inflammation in the colonic mucosa and altering the intestinal microenvironment [2,4].

Malignant tumors require substantial metabolic resources to sustain continuous cell division and proliferation [5]. Unlike normal cells, tumor cells exhibit the Warburg effect, generating energy through glycolysis even under oxygen-sufficient conditions while simultaneously reprogramming amino acid and lipid metabolic pathways to support biomolecular synthesis [5,6]. Therefore, the host’s dietary intake can significantly influence tumor growth and proliferation by directly supplying metabolites and nutrients to the tumor microenvironment or by inducing systemic endocrine and metabolic changes [2,5]. In particular, the composition and source of dietary macronutrients may act as important external factors that regulate tumor energy metabolism [2].

Recently, the gut microbiome has emerged as an important mediator linking dietary factors to CRC pathogenesis [1,4]. Trillions of microorganisms residing in the gastrointestinal tract play essential roles in host nutrient metabolism, immune system regulation, pathogen inhibition, and the maintenance of intestinal mucosal barrier integrity [1,2,7]. Diet is a major environmental factor that determines the diversity and taxonomic composition of the gut microbiome [1]. Microbial metabolism of dietary components can generate carcinogenic substances, and microbial modification of dietary constituents may contribute to the development of CRC [8]. In healthy conditions, major phyla such as Firmicutes and Bacteroidetes maintain microbial homeostasis and produce beneficial short-chain fatty acids, which provide energy to intestinal epithelial cells and exert anti-inflammatory effects [3,6].

However, prolonged exposure to specific dietary conditions can disrupt the gut microbial ecosystem, leading to dysbiosis [2]. High-fat diets can alter bile acid metabolism, impair intestinal barrier integrity, and promote expansion of inflammation-associated microbial taxa, thereby contributing to colorectal carcinogenesis [9]. In addition, excessive intake of carbohydrates or specific fatty acids may promote the proliferation of pathogenic bacteria and increase intestinal permeability, resulting in an enhanced influx of endotoxins into circulation [2]. In particular, under chronic inflammatory conditions, an increased abundance of opportunistic pathogens, such as Proteobacteria, is frequently observed and has been closely associated with inflammatory bowel disease and CRC development [3,9].

Although increasing evidence supports complex interactions between diet, the gut microbiome, and CRC, the differential impacts of specific macronutrient compositions within diets remain unclear [1,2]. In particular, metabolic and microbial alterations induced by excessive carbohydrate intake, as well as differences in dietary fat sources, such as animal-derived saturated fat (lard) versus plant-derived saturated fat (coconut oil), have not been fully elucidated. Although both diets are rich in saturated fat, lard and coconut oil differ substantially in terms of fatty acid composition, particularly in the proportions of long- and medium-chain fatty acids, which may differentially influence host metabolism, bile acid metabolism, inflammatory responses, and gut microbial ecology [10,11]. These findings highlight the need for a precise nutritional approach that considers not only caloric intake but also the composition and source of dietary macronutrients.

Therefore, this study aimed to investigate how different dietary patterns are associated with CRC development and gut microbiome alterations in an azoxymethane (AOM)/dextran sulfate sodium (DSS)-induced mouse model. Tumor development, morphologic inflammatory cell infiltration, and temporal changes in gut microbial communities were comparatively analyzed in mice fed a standard diet (SD), high-carbohydrate diet (HCD), lard-based low-carbohydrate high-fat diet (HFL), or coconut oil-based low-carbohydrate high-fat diet (HFC) during AOM/DSS-induced colorectal carcinogenesis. Because the experimental diets differed in more than one macronutrient feature, the design was intended to compare dietary patterns and not to isolate the independent effects of carbohydrate restriction, total fat, or fat source.

## 2. Results

### 2.1. Effects of Diets on Body Weight, Colon Length, and Splenic Weight During AOM/DSS-Induced Colorectal Carcinogenesis

**Figure 1:**
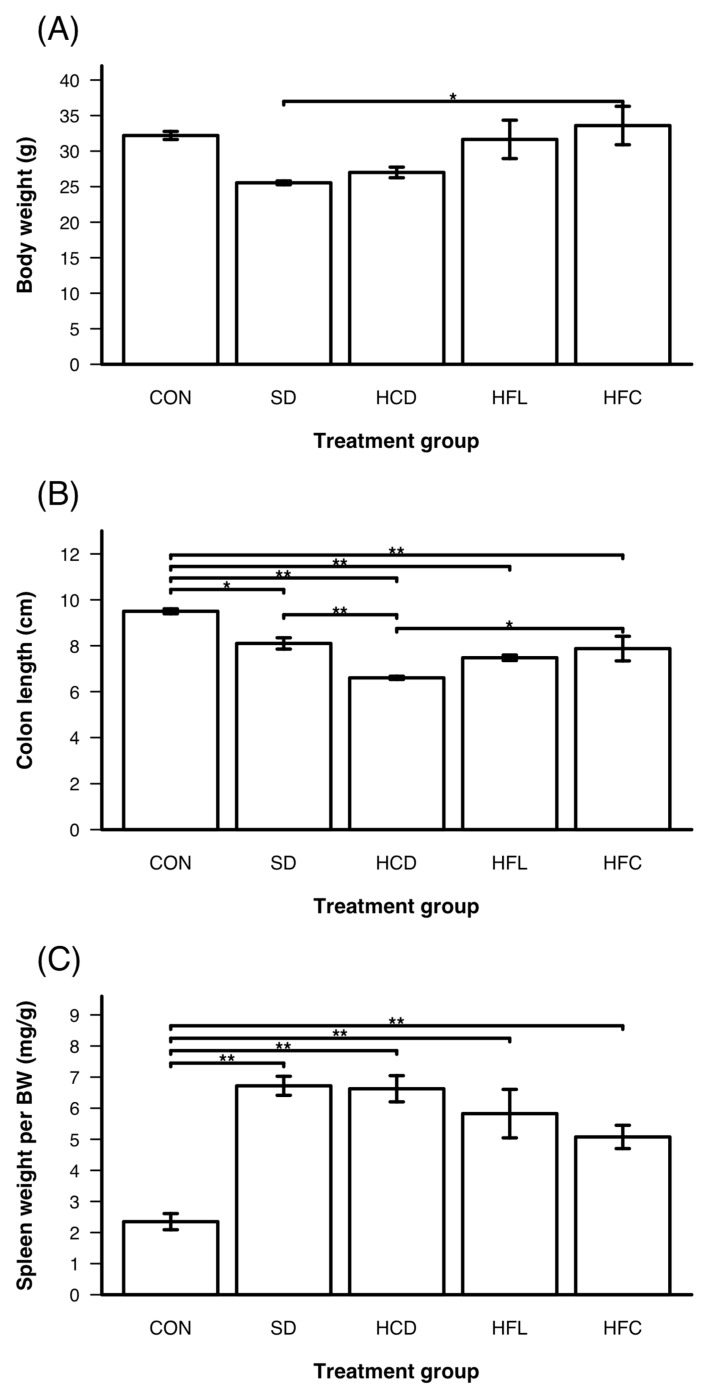
Effects of dietary composition on body weight, colon length, and splenic weight during AOM/DSS-induced colorectal carcinogenesis. Body weight (**A**), colon length (**B**), and splenic weight normalized to body weight (**C**) were measured on day 72 following dietary intervention. CON, control group (no AOM/DSS treatment with standard diet); SD, standard diet group; HCD, high-carbohydrate diet group; HFL, low-carbohydrate high-fat lard-based diet group; HFC, low-carbohydrate high-fat coconut oil-based diet group. Mice were allocated as follows: CON, *n* = 4; SD, HCD, HFL, and HFC, *n* = 6 each. The analyzed sample size differed among endpoints because of unavailable measurements; each dot represents an individual mouse, not a technical replicate. Data are presented as means ± SEM. Statistical comparisons were performed using one-way ANOVA followed by Tukey’s multiple-comparison test. * *p* < 0.05, ** *p* < 0.01.

The effects of different dietary interventions on body weight, colon length, and splenic weight were evaluated in mice during 72 days of AOM/DSS-induced colorectal carcinogenesis. One-way ANOVA showed an overall diet-group effect on final body weight (*p* = 0.011). In Tukey-adjusted post hoc comparisons, the HFC group had a higher final body weight than the SD group (*p* = 0.021), whereas other pairwise differences did not remain statistically significant after correction (Figure 1A).

Colonic length also differed among groups (ANOVA *p* < 0.001) (Figure 1B). All AOM/DSS-treated groups showed shorter colons than the control (CON) group in Tukey-adjusted comparisons. Among AOM/DSS-treated groups, the HCD group showed the greatest shortening, and colon length was longer in the HFC group than in the HCD group (*p* = 0.037).

Splenic weight normalized to body weight differed among the AOM/DSS-treated diet groups (ANOVA *p* = 0.002) (Figure 1C). In Tukey-adjusted comparisons, the HCD group had a higher normalized splenic weight than the SD and HFC groups, whereas the HFC group had a lower value than the HFL group.

Collectively, these findings indicate that dietary composition was associated with physiological differences during AOM/DSS-induced colorectal carcinogenesis, but several pairwise differences were attenuated after correction for multiple comparisons.

### 2.2. Effects of Diets on Colon Cancer Development During AOM/DSS-Induced Colorectal Carcinogenesis

**Figure 2:**
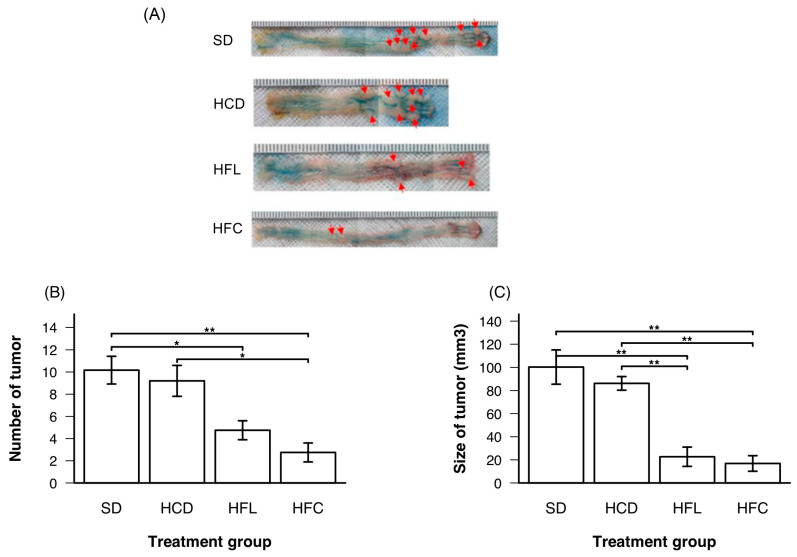
Effects of dietary composition on macroscopic colon tumor burden during AOM/DSS-induced colorectal carcinogenesis. (**A**) Representative macroscopic images of colons from each experimental group on day 72 after AOM/DSS induction. Red arrows indicate colonic tumors. (**B**) Number of tumors per mouse in each experimental group. (**C**) Total tumor size per mouse in each experimental group. SD, standard diet group; HCD, high-carbohydrate diet group; HFL, low-carbohydrate high-fat lard-based diet group; HFC, low-carbohydrate high-fat coconut oil-based diet group. Mice were allocated at *n* = 6 per AOM/DSS-treated group; analyzed n values were SD, *n* = 5–6; HCD, *n* = 4–5; HFL, *n* = 4; and HFC, *n* = 4, depending on the endpoint. Each dot represents an individual mouse. Data are presented as means ± SEM. Statistical comparisons were performed using one-way ANOVA followed by Tukey’s multiple-comparison test. * *p* < 0.05, ** *p* < 0.01.

To investigate the effects of dietary composition on colon tumor development, tumor multiplicity and size were evaluated in mice subjected to AOM/DSS-induced colorectal carcinogenesis after 72 days of dietary intervention. Macroscopic examination of the colon revealed multiple tumors in all AOM/DSS-treated groups (Figure 2A). However, the extent of tumor development differed according to dietary conditions, with visibly fewer tumors observed in the HFL and HFC groups than in the SD and HCD groups.

Quantitative analysis showed an overall diet-group effect on tumor number (ANOVA *p* = 0.002) (Figure 2B). In Tukey-adjusted comparisons, tumor number was lower in the HFC group than in the SD and HCD groups, and lower in the HFL group than in the SD group; the HFL-HCD comparison did not remain statistically significant after correction. Total tumor size also differed among groups (ANOVA *p* < 0.001), with lower values in the HFL and HFC groups than in the SD and HCD groups after Tukey correction (Figure 2C).

Collectively, these findings indicate that low-carbohydrate high-fat diets were associated with reduced macroscopic tumor multiplicity and overall tumor burden in this model. Because lesion grade was not histologically classified in the present analysis, these macroscopic findings should be interpreted as tumor-burden measurements rather than definitive evidence of altered histological progression.

### 2.3. Effects of Diets on H&E-Based Inflammatory Cell Infiltration During AOM/DSS-Induced Colorectal Carcinogenesis

**Figure 3:**
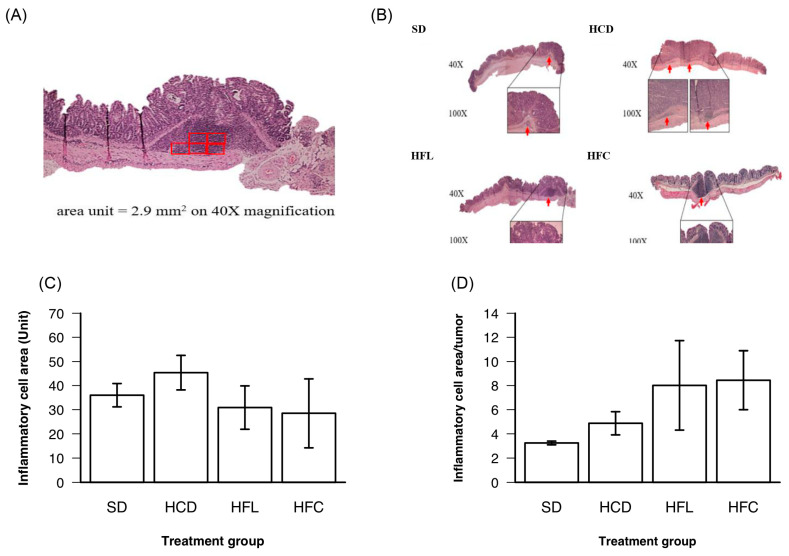
Effects of dietary composition on H&E-based inflammatory cell infiltration in tumor-bearing colonic tissue during AOM/DSS-induced colorectal carcinogenesis. (**A**) Representative H&E-stained image demonstrating the method used to quantify inflammatory cell infiltration area. Red rectangles indicate the representative unit areas used for inflammatory cell infiltration quantification (2.9 mm^2^ each). One unit was defined as an area of 2.9 mm^2^ at ×40 magnification under an optical microscope. (**B**) Representative H&E-stained images of tumor tissue and surrounding areas from each experimental group at ×40 and ×100 magnification showing lymphocyte-like inflammatory cell infiltration (red arrows). (**C**) Total inflammatory cell infiltration area within tumor-bearing colonic tissue in each experimental group. (**D**) Inflammatory cell infiltration area normalized per tumor in each experimental group. SD, standard diet group; HCD, high-carbohydrate diet group; HFL, low-carbohydrate high-fat lard-based diet group; HFC, low-carbohydrate high-fat coconut oil-based diet group. Analyzed n values were SD, *n* = 5; HCD, *n* = 4; HFL, *n* = 4; and HFC, *n* = 4. Data are presented as means ± SEM. Statistical comparisons were performed using one-way ANOVA followed by Tukey’s multiple-comparison test. H&E staining does not define immune-cell subsets.

To assess the effects of dietary composition on inflammatory cell infiltration within tumor-bearing tissues, H&E-stained colonic sections were analyzed after 72 days of dietary intervention. Because H&E staining does not distinguish CD8+ T cells, CD4+ T cells, B cells, regulatory T cells, macrophages, or other immune cell populations, the quantified area was interpreted as morphologic lymphocyte-like inflammatory cell infiltration rather than a defined antitumor lymphocyte response (Figure 3A,B).

Quantitative analysis showed that the total inflammatory cell infiltration area within tumor-bearing colonic tissue varied among groups, with the numerically largest value observed in the HCD group (Figure 3C). Because the HFL and HFC groups had fewer tumors, normalization by tumor number can inflate per-tumor infiltration values. We therefore present both total infiltration area and per-tumor infiltration area and interpret the latter as an exploratory normalization. After one-way ANOVA with correction for multiple comparisons, the per-tumor infiltration values did not show statistically significant differences among diet groups (Figure 3D).

These findings indicate that H&E-based inflammatory cell infiltration should be interpreted cautiously because it reflects both tumor burden and morphologic inflammatory changes. Additional normalization to tumor area, mucosal area, or lesion grade, together with immunophenotyping, would be required to determine whether the infiltrate represents antitumor immunity or nonspecific inflammation.

### 2.4. Effects of Diets on Gut Microbial Composition During AOM/DSS-Induced Colorectal Carcinogenesis

**Figure 4:**
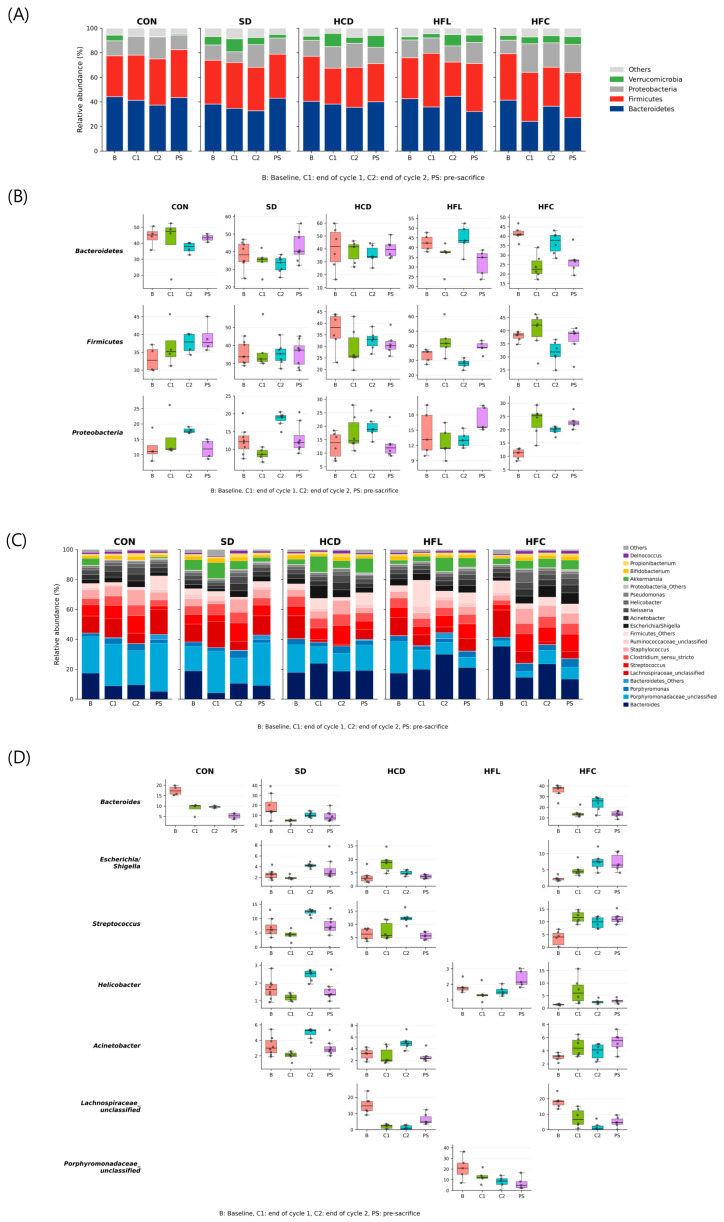
Effects of dietary composition on gut microbial composition during AOM/DSS-induced colorectal carcinogenesis. (**A**) Relative abundance of major bacterial phyla at four time points during the experimental period in each experimental group. (**B**) Temporal alterations in the relative abundance of major bacterial phyla, including Bacteroidetes, Firmicutes, and Proteobacteria, in each experimental group. (**C**) Relative abundance of major bacterial genera at four time points during the experimental period in each experimental group. (**D**) Temporal alterations in selected bacterial genera, including Bacteroides, Escherichia/Shigella, Streptococcus, Helicobacter, Acinetobacter, Lachnospiraceae_unclassified, and Porphyromonadaceae_unclassified, in each experimental group. B, baseline; C1, end of the first DSS cycle; C2, end of the second DSS cycle; PS, pre-sacrifice. CON, control group; SD, standard diet group; HCD, high-carbohydrate diet group; HFL, low-carbohydrate high-fat lard-based diet group; HFC, low-carbohydrate high-fat coconut oil-based diet group. Each sample represents fecal material from an individual mouse at the indicated time point, not a technical replicate. Group sizes varied by available fecal samples across time points.

To investigate whether different dietary compositions influenced the gut microbial composition during CRC development, a phylum-level microbiome analysis was performed in mice subjected to AOM/DSS treatment under various dietary conditions (Figure 4A,B). Compared with the CON group, the AOM/DSS-treated groups exhibited temporal alterations in gut microbial composition, and these changes were more pronounced in the low-carbohydrate high-fat diet groups, particularly the HFL and HFC groups. In the HFL and HFC groups, the relative abundance of Bacteroidetes showed substantial temporal variation and tended to remain lower at later time points, whereas Proteobacteria exhibited a relatively higher abundance during tumor progression, particularly in the HFC group. Firmicutes also exhibited temporal fluctuations across experimental groups. In contrast, the CON group maintained relatively stable proportions of major bacterial phyla throughout the study period. Because microbiome data were obtained from a small exploratory cohort and repeated-measures modeling was not robustly applicable, these longitudinal patterns are presented descriptively. Collectively, these findings suggest that low-carbohydrate high-fat dietary patterns are associated with altered phylum-level gut microbial composition during AOM/DSS-induced colorectal carcinogenesis.

Genus-level gut microbiome analysis further revealed distinct microbial alterations associated with dietary treatment in AOM/DSS-induced colorectal carcinogenesis (Figure 4C,D). Compared with the CON group, the AOM/DSS-treated groups exhibited temporal shifts in bacterial composition, with the most pronounced changes observed in the HFC group. In the HFC group, genera belonging to Proteobacteria, including Escherichia/Shigella, Acinetobacter, and Helicobacter, tended to increase at later time points during tumor progression. In contrast, Bacteroides and Lachnospiraceae_unclassified showed relatively lower abundances at later stages in the HFC group. Inflammation-associated genera, such as Streptococcus and Clostridium sensu stricto, also showed temporal fluctuations in the low-carbohydrate high-fat diet groups. Similar but less prominent compositional changes were observed in the HFL group, including alterations in Helicobacter and Porphyromonadaceae_unclassified. These taxa-level findings should be interpreted cautiously because V3-V4 16S rRNA sequencing generally provides limited taxonomic resolution and does not establish species-level pathogenicity or microbial function. Overall, these findings indicate that dietary pattern is associated with altered gut microbial dynamics during AOM/DSS-induced colorectal carcinogenesis.

### 2.5. Effects of Diets on Gut Microbial Diversity During AOM/DSS-Induced Colorectal Carcinogenesis

**Figure 5:**
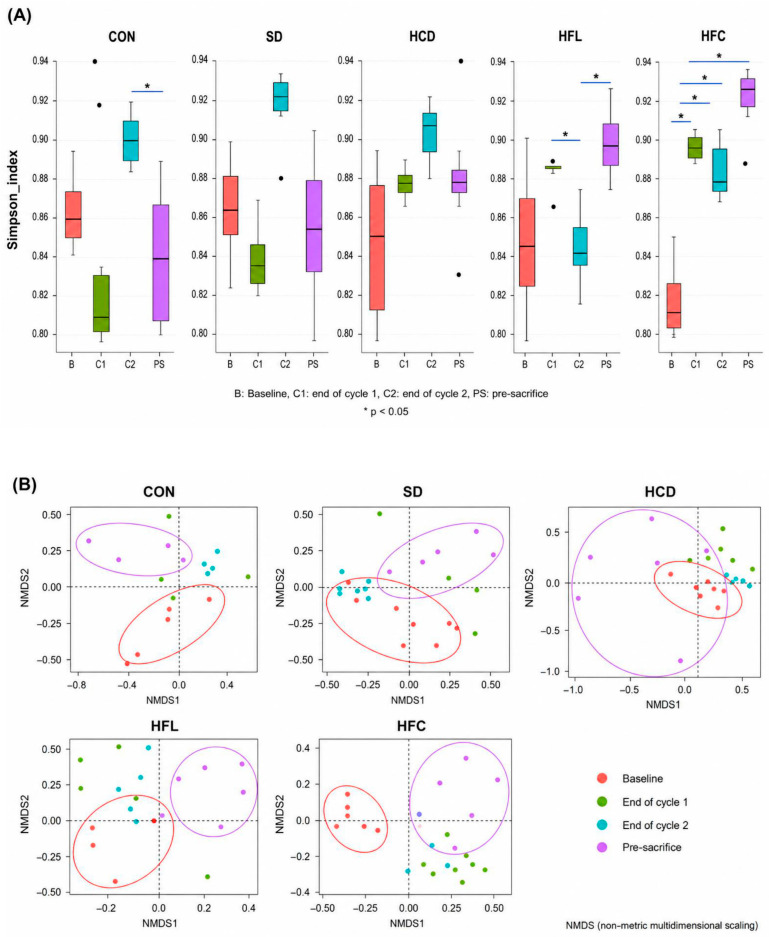
Analysis of gut microbial diversity during AOM/DSS-induced colorectal carcinogenesis according to dietary composition. (**A**) Alpha diversity analysis using the Simpson index at four time points during the experimental period. (**B**) Beta diversity analysis using non-metric multidimensional scaling (NMDS) demonstrates temporal alterations in microbial community structure in each experimental group. B, baseline; C1, end of the first DSS cycle; C2, end of the second DSS cycle; PS, pre-sacrifice. CON, control group; SD, standard diet group; HCD, high-carbohydrate diet group; HFL, low-carbohydrate high-fat lard-based diet group; HFC, low-carbohydrate high-fat coconut oil-based diet group. Each point represents a fecal sample from an individual mouse at the indicated time point, not a technical replicate. Group sizes varied by available fecal samples across time points. Asterisks in panel A indicate nominal, unadjusted pairwise comparisons at individual time points and are provided only as descriptive markers. Because of the small sample size and incomplete longitudinal matching, no formal repeated-measures or diet-by-time interaction analysis was performed.

Alpha diversity analysis using the Simpson index demonstrated temporal alterations in gut microbial diversity during AOM/DSS-induced colorectal carcinogenesis according to dietary intervention (Figure 5A). The CON group exhibited moderate fluctuations in microbial diversity throughout the experimental period. In contrast, the AOM/DSS-treated groups, particularly the HFL and HFC groups, exhibited more distinct temporal changes in alpha diversity. The HFC group showed higher Simpson index values at later time points, and the HFL group also showed temporal variation in microbial diversity. In contrast, the SD and HCD groups showed less pronounced changes over time. Because repeated-measures analysis was limited by small sample size and incomplete longitudinal matching, these diversity results are interpreted as descriptive temporal patterns rather than definitive diet-by-time interaction effects. Collectively, these findings suggest that dietary pattern is associated with alterations in gut microbial diversity during AOM/DSS-induced colorectal carcinogenesis.

Beta diversity analysis using non-metric multidimensional scaling (NMDS) demonstrated distinct clustering patterns of the gut microbiome according to dietary intervention during AOM/DSS-induced colorectal carcinogenesis (Figure 5B). Temporal shifts in the microbial community structure were observed across experimental groups, with a relatively greater separation between the early and late time points in the HFL and HFC groups. In particular, the HFC group demonstrated clearer clustering of baseline and pre-euthanasia samples than the CON and SD groups, suggesting greater temporal divergence in microbial community composition. In contrast, the SD group showed a substantial overlap between the time points, indicating a relatively limited community separation over time. These findings suggest that low-carbohydrate high-fat dietary patterns are associated with altered gut microbial community structure, although formal longitudinal diet-by-time interaction testing was not performed.

## 3. Discussion

This study investigated the association of different dietary patterns with macroscopic tumor burden, morphologic inflammatory responses, and alterations in the gut microbiome in an AOM/DSS-induced mouse model of colitis-associated colorectal cancer. Our findings suggest that dietary pattern is associated with differences in tumor burden and intestinal microbial ecology during AOM/DSS-induced colorectal tumorigenesis; however, the modest sample size, non-isocaloric diet design, and simultaneous changes in carbohydrate content, fat content, fat source, and caloric density limit causal interpretation.

Consistent with previous studies, AOM/DSS treatment induced marked physiological alterations, including body weight reduction, colon shortening, and splenic enlargement, indicating severe intestinal inflammation and systemic immune activation during colorectal carcinogenesis [12,13]. The HFL and HFC diets changed several nutritional variables simultaneously, including lower carbohydrate content, higher fat content, different fat sources, and higher caloric density than the SD and HCD diets. Food intake was not measured. Therefore, differences in body weight and tumor burden may reflect not only macronutrient composition but also differences in total caloric intake, palatability, and energy balance. Pair-feeding, isocaloric dietary design, and additional normal-carbohydrate high-fat or single-macronutrient-adjusted diet groups would be required to determine whether carbohydrate restriction, increased total fat, or fat source independently affects colorectal tumorigenesis.

Cancer cells depend heavily on glucose metabolism to sustain their rapid proliferation and survival. The Warburg effect describes the metabolic reprogramming of cancer cells toward aerobic glycolysis even under oxygen-sufficient conditions [14,15]. Colorectal cancer cells are highly dependent on glucose metabolism and undergo substantial metabolic adaptations to support their proliferation and survival [14,16]. The higher tumor burden in the HCD group may be consistent with a metabolic environment permissive for tumor growth, but this interpretation remains speculative because metabolic parameters and food intake were not measured. Hyperglycemia and insulin signaling are also known to activate proliferative pathways, including PI3K/AKT/mTOR signaling, which contributes to colorectal carcinogenesis [17,18]. Therefore, the enhanced tumor development observed in the HCD group may reflect metabolic conditions that are favorable for tumor cell proliferation. In contrast, the relatively reduced tumor development observed in the HFL and HFC groups suggests that high-fat diets may differentially affect tumor progression depending on the metabolic context. Although high-fat diets are generally associated with obesity, chronic inflammation, and increased cancer risk, several studies have suggested that reduced carbohydrate availability may suppress glucose-dependent tumor growth under certain conditions [19]. In the present study, the low-carbohydrate and high-fat diet groups exhibited increased body weight and persistent inflammatory changes; however, tumor formation was lower than that in the HCD group. These findings indicate that obesity-associated metabolic alterations alone may not directly correlate with tumor development and that the balance between carbohydrate and lipid metabolism may play a critical role in colorectal carcinogenesis.

The colon shortening observed in all AOM/DSS-treated groups further supports the presence of chronic intestinal inflammation and epithelial injury. DSS-induced colitis is known to disrupt epithelial barrier integrity, promote inflammatory cytokine production, and activate NF-kB and STAT3 signaling pathways, thereby facilitating tumorigenesis [12,13]. Despite differences in tumor burden, all dietary intervention groups showed shortened colons compared to controls, indicating that chronic inflammation persisted throughout tumor development. Spleen enlargement was also observed in all the AOM/DSS-treated groups, reflecting systemic immune activation during tumor development. In the present study, H&E staining permitted morphologic assessment of inflammatory cell infiltration but did not define immune-cell subsets or distinguish antitumor lymphocyte immunity from nonspecific inflammatory infiltration. Moreover, normalization by tumor number may overestimate per-tumor infiltration in groups with fewer tumors. Therefore, immunohistochemistry or flow cytometric analysis of immune populations, together with normalization to tumor area, mucosal area, and lesion grade, will be necessary to clarify the immunological significance of these findings.

Another important finding of the present study was that dietary pattern substantially influenced the gut microbial ecology during AOM/DSS-induced colorectal carcinogenesis. The HFL and HFC groups demonstrated greater temporal variability in microbial composition than the CON and SD groups. At the phylum level, fluctuations in Bacteroidetes and the relative expansion of Proteobacteria became more evident during tumor progression in the low-carbohydrate high-fat diet groups, with the most prominent changes observed in the HFC group. An increased abundance of Proteobacteria has been frequently associated with altered intestinal microbial ecology and inflammatory conditions in both experimental colitis and colorectal cancer models [4,20,21]. However, the present 16S rRNA data do not establish whether these compositional changes caused, opposed, or merely accompanied tumor development.

At the genus level, several inflammation-associated or potentially pathogenic taxa, including Escherichia/Shigella, Acinetobacter, Helicobacter, and Streptococcus, exhibited temporal increases in the low-carbohydrate high-fat diet groups. Similar microbial alterations have been implicated in epithelial barrier dysfunction, inflammatory signaling, and colorectal carcinogenesis [22,23]. These findings suggest that intestinal microbial homeostasis was disrupted during chronic inflammatory tumorigenesis. However, the coexistence of more pronounced dysbiosis with lower macroscopic tumor burden in the HFL and HFC groups is a key paradox of the present study. One possible explanation is that reduced carbohydrate availability may have limited glucose-dependent tumor growth strongly enough to outweigh the potentially pro-carcinogenic effect of dysbiosis. A second possibility is that coconut oil-derived medium-chain fatty acids, including lauric acid, may exert direct antimicrobial, immunomodulatory, or antitumor effects. A third possibility is that high-fat low-carbohydrate feeding may promote ketogenesis, and ketone bodies have been proposed to influence tumor metabolism under some conditions. In addition, morphologic inflammatory cell infiltration may reflect immune activation within the tumor-bearing mucosa; however, without immune-cell phenotyping, we cannot determine whether this represents antitumor immunity or nonspecific inflammation. These possibilities support an integrated hypothesis in which dietary regulation of colorectal tumorigenesis reflects the combined effects of host metabolism, immune activation, and microbial ecology, rather than microbial composition alone. Therefore, tumor risk cannot be inferred solely from the abundance of inflammation-associated microbial taxa.

In addition, alpha- and beta-diversity analyses suggested greater temporal divergence in microbial communities in the low-carbohydrate high-fat diet groups during colorectal carcinogenesis. The HFC group exhibited the most distinct clustering pattern and temporal separation in the NMDS analysis, suggesting a substantial restructuring of microbial community composition over time. Coconut oil is enriched in medium-chain fatty acids, particularly lauric acid, which has been reported to exert antimicrobial, immunomodulatory, and potential antitumor effects [10,11]. Thus, the distinct microbial dynamics observed in the HFC group may reflect not only a low-carbohydrate high-fat dietary pattern but also biological effects specific to coconut oil-derived fatty acids. This possibility should be tested directly in future studies.

Accumulating evidence suggests that the gut microbiome contributes to colorectal carcinogenesis by modulating epithelial barrier integrity, microbial metabolite production, bile acid metabolism, and mucosal immune responses [4,24,25]. In the present study, however, short-chain fatty acids, bile acid profiles, serum LPS, tight-junction proteins, and other barrier-function indicators were not measured, and functional prediction analysis such as PICRUSt2 was not performed. Therefore, any links between the observed microbiome shifts and microbial metabolites, bile acid metabolism, epithelial barrier function, or tumor-promoting pathways remain speculative. Further mechanistic studies integrating metagenomics or functional prediction, metabolomics, barrier-function assays, and host-microbiome interaction analysis are necessary to clarify the precise role of diet-associated microbial dysbiosis in colorectal tumorigenesis.

This study had several limitations. First, the sample size in each experimental group was relatively small, and some endpoint analyses included fewer animals because of unavailable measurements. Second, the HFL and HFC diets simultaneously changed carbohydrate proportion, fat proportion, fat source, and caloric density; therefore, the present study evaluates low-carbohydrate high-fat dietary patterns and cannot disentangle the independent effects of carbohydrate restriction, increased total fat, or lard versus coconut oil. Third, food intake was not measured, and pair-feeding or isocaloric dietary control was not performed. Fourth, formal repeated-measures modeling of longitudinal body-weight and microbiome data was limited by the available dataset and sample size; therefore, longitudinal microbiome results were interpreted descriptively and diet-by-time interaction effects were not established. Fifth, histological lesion grading, including inflammation severity, dysplasia, adenoma, adenocarcinoma, and tumor grade, was not included; therefore, macroscopic tumor number and size cannot fully establish effects on histological colorectal carcinogenesis. Sixth, H&E-based infiltration analysis does not identify immune-cell subsets, and additional immunohistochemistry would be required to distinguish antitumor lymphocyte immunity from inflammatory infiltration. Seventh, tumor counting and measurement were performed using predefined gross criteria, but blinding was not documented in the original experimental record. Eighth, metabolic and inflammatory mediators, including serum glucose, insulin, triglycerides, cholesterol, ketone bodies, systemic or colonic cytokines, fecal bile acids, short-chain fatty acids, serum LPS, and intestinal barrier markers, were not measured. Ninth, the microbiome analysis was performed using V3-V4 16S rRNA sequencing, which limits species-level taxonomic assignment and functional or metabolomic interpretation; functional prediction analysis such as PICRUSt2 was not performed. Finally, only the standard-diet control group was maintained without AOM/DSS, and non-AOM/DSS controls for HCD, HFL, and HFC diets were not included; this limits interpretation of diet-specific microbiome changes independent of carcinogen/colitis treatment. Therefore, additional mechanistic studies using isocaloric or pair-fed designs, single-macronutrient-controlled diet groups, repeated-measures statistical designs, and integrated histopathological scoring, metagenomics, metabolomics, barrier-function testing, metabolic profiling, and immune phenotyping are required.

Taken together, the present study suggests that low-carbohydrate high-fat dietary patterns are associated with differences in macroscopic tumor burden, morphologic inflammatory cell infiltration, and gut microbial ecology in an AOM/DSS-induced colorectal cancer model. High-carbohydrate dietary conditions were associated with greater tumor burden, whereas low-carbohydrate high-fat diets were associated with altered microbial community structure and lower macroscopic tumor burden under the present experimental conditions. Because of the non-isocaloric design, simultaneous macronutrient changes, small sample size, absence of histological lesion grading, lack of repeated-measures interaction testing, and limited immune, metabolic, metabolomic, and barrier-function profiling, these findings should be considered exploratory and hypothesis-generating. Further mechanistic studies investigating host metabolism, immune responses, host-microbiome interactions, and microbiome-derived metabolites are needed to define how dietary patterns modulate colorectal cancer progression.

## 4. Materials and Methods

### 4.1. Mice

Ten-week-old male C57BL/6 mice were purchased from KOATEC (Pyeongtaek, Gyeonggi-do, Republic of Korea). The mice were housed in a specific pathogen-free (SPF) facility under controlled environmental conditions with free access to drinking water and a commercial pellet diet for 1 week prior to the experiments. All experimental procedures were approved by the Institutional Animal Care and Use Committee of Kosin University College of Medicine (approved number: KMAP-17-13) and performed in accordance with the institutional guidelines for animal care and use.

### 4.2. Colitis-Associated Tumor Induction and Dietary Intervention

Twenty-eight mice were allocated into five groups: a CON group (no AOM/DSS treatment with a standard diet, *n* = 4), a SD group (AOM/DSS treatment with a standard diet, *n* = 6), a HCD group (AOM/DSS treatment with a high-carbohydrate diet, *n* = 6), a HFL group (AOM/DSS treatment with a low-carbohydrate high-fat lard-based diet, *n* = 6), and a HFC group (AOM/DSS treatment with a low-carbohydrate high-fat coconut oil-based diet, *n* = 6). The detailed nutrient composition of each experimental diet is summarized in Table 1. The HFL and HFC diets simultaneously differed from the SD and HCD diets in carbohydrate proportion, fat proportion, fat source, and caloric density (SD, 3.1 kcal/g; HCD, 3.85 kcal/g; HFL and HFC, 5.1 kcal/g). Food intake was not recorded, and the study was not designed as a pair-fed, isocaloric, or single-macronutrient-controlled experiment. Dietary interventions were initiated simultaneously with AOM administration and maintained until euthanasia on day 72.

To induce colitis-associated colon cancer, the mice received a single intraperitoneal injection of AOM (10 mg/kg body weight) (Sigma-Aldrich, St. Louis, MO, USA), followed by three cycles of DSS. The first cycle consisted of 1.5% DSS in drinking water for 1 week, followed by regular water for 2 weeks. The second and third cycles consisted of 2% DSS for 1 week, followed by 2 weeks of regular water.

**Figure 6:**
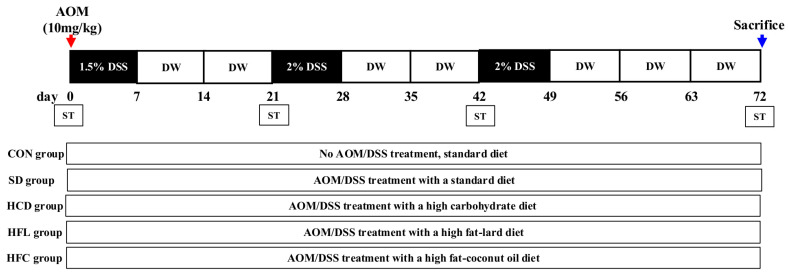
Experimental design of AOM/DSS-induced colitis-associated colorectal carcinogenesis and dietary intervention. Mice received a single intraperitoneal injection of azoxymethane (AOM) (10 mg/kg body weight) on day 0, followed by three cycles of dextran sulfate sodium (DSS). The first cycle consisted of 1.5% DSS for 7 days, followed by distilled water (DW) for 14 days. The second and third cycles consisted of 2.0% DSS for 7 days, followed by DW for 14 days and 21 days, respectively. Dietary interventions were initiated simultaneously with AOM administration and maintained until euthanasia on day 72. Stool samples were collected at four time points during the experimental period. CON, control group, *n* = 4; SD, standard diet, *n* = 6; HCD, high-carbohydrate diet, *n* = 6; HFL, low-carbohydrate high-fat lard-based diet, *n* = 6; HFC, low-carbohydrate high-fat coconut oil-based diet, *n* = 6; ST, stool collection time.

During the experimental period, stool samples were collected at four time points: before AOM administration, at the end of the first DSS cycle, at the end of the second DSS cycle, and immediately before euthanasia. Body weight, colon length, and splenic weight were also measured. After euthanasia, the colon was longitudinally opened to determine the number of macroscopic tumors, and tumor size was measured using a caliper. Tumor counting and measurement were performed using predefined gross criteria; blinding was not documented in the original experimental record and is therefore acknowledged as a limitation. The experimental design and timeline are shown in Figure 6.

### 4.3. Histopathological Analysis

To evaluate tumor-associated tissue morphology and inflammatory cell infiltration, tumor and adjacent tissues were collected, fixed in 10% neutral-buffered formalin, and embedded in paraffin. Tissue sections (4–6 um thick) were prepared and stained with hematoxylin and eosin (H&E). Histopathological examination was performed under a light microscope to assess morphologic lymphocyte-like inflammatory cell infiltration around the tumors. The extent of inflammatory cell infiltration was quantified by measuring the infiltration area, with one unit defined as an area of 2.9 mm^2^ at ×40 magnification. Formal histological classification of lesions, including inflammation score, dysplasia, adenoma, adenocarcinoma, and tumor grade, was not performed in the present analysis.

### 4.4. Gut Microbiome Analysis

To evaluate the gut microbiome, stool samples were collected in sterile tubes from each mouse at four time points: before AOM administration, at the end of the first and second DSS cycles, and immediately before euthanasia. All samples were immediately stored at −80 °C until analysis. After completion of sample collection, the stool samples were transported to Bioeleven Co., Ltd. (Seoul, Republic of Korea) for sequencing. Total bacterial DNA was extracted from the stool samples using a QIAamp Fast DNA Stool Mini Kit (Qiagen, Hilden, Germany) according to the manufacturer’s instructions. The concentration and purity of extracted DNA were assessed using a NanoDrop spectrophotometer (Thermo Fisher Scientific, Waltham, MA, USA). The V3–V4 region of the bacterial 16S rRNA gene was amplified by polymerase chain reaction (PCR), and the amplified products were purified using AMPure XP magnetic beads (Beckman Coulter, High Wycombe, UK). The quality and size of purified PCR products were evaluated using an Agilent Bioanalyzer (Agilent Technologies, Santa Clara, CA, USA). Sequencing libraries were prepared according to the Illumina 16S Metagenomic Sequencing Library Preparation protocol and sequenced on an Illumina MiSeq platform (Illumina, San Diego, CA, USA). Raw sequencing data were processed for quality filtering and taxonomic assignment to evaluate the differences in gut microbial composition among the experimental groups. This 16S rRNA approach was used for compositional profiling and does not provide direct species-level confirmation, microbial metabolite measurements, barrier-function assessment, or functional pathway prediction.

### 4.5. Statistical Analyses

Data are presented as means ± standard errors of the mean (SEM). Statistical analyses were performed using GraphPad Prism version 10.0.0 (GraphPad Software, San Diego, CA, USA) and rechecked using R version 4.2.2 (R Foundation for Statistical Computing, Vienna, Austria). For endpoint comparisons among multiple diet groups, one-way analysis of variance (ANOVA) was performed followed by Tukey’s multiple-comparison test when the global ANOVA *p*-value was < 0.05. Only final body weight was used for formal endpoint comparison. Longitudinal body-weight and microbiome data were interpreted descriptively because the small cohort size, missing samples, and exploratory 16S design limited robust repeated-measures ANOVA or mixed-effects modeling for diet, time, and diet-by-time interaction effects. Accordingly, longitudinal findings are reported as temporal patterns rather than definitive interaction effects. Statistical significance was set at a *p*-value < 0.05. The exact analyzed sample size is provided in the corresponding figure legends when it differs from the number of animals allocated to each group.

## Figures and Tables

**Table 1:** Nutrient compositions in diets.

Composition	SD		HCD		HFL		HFC	
	gm%	Kcal%	gm%	Kcal%	gm%	Kcal%	gm%	Kcal%
Protein	18.6	24	19.2	20	23.5	18.3	23.5	18.3
Carbohydrate	44.2	58	67.3	70	27.3	21.4	27.3	21.4
Fat	6.2	18	4.3	10	34.3	60.3	34.3	60.3
Total		100		100		100		100
Kcal/gm	3.1		3.85		5.1		5.1	

SD, standard diet; HCD, high-carbohydrate diet; HFL, low-carbohydrate high-fat lard-based diet; HFC, low-carbohydrate high-fat coconut oil-based diet.

## Data Availability

The technical appendix and dataset are available from the corresponding author.

## Abbreviations

The following abbreviations are used in this manuscript:

AOM	Azoxymethane
CRC	Colorectal cancer
DSS	Dextran sulfate sodium
CON	Control group
SD	Standard diet
HCD	High-carbohydrate diet
HFL	Low-carbohydrate high-fat lard-based diet
HFC	Low-carbohydrate high-fat coconut oil-based diet
H&E	Hematoxylin and eosin
NMDS	Non-metric multidimensional scaling
SEM	Standard error of the mean
SPF	Specific pathogen-free
